# mobileOG-db: a Manually Curated Database of Protein Families Mediating the Life Cycle of Bacterial Mobile Genetic Elements

**DOI:** 10.1128/aem.00991-22

**Published:** 2022-08-29

**Authors:** Connor L. Brown, James Mullet, Fadi Hindi, James E. Stoll, Suraj Gupta, Minyoung Choi, Ishi Keenum, Peter Vikesland, Amy Pruden, Liqing Zhang

**Affiliations:** a Department of Genetics, Bioinformatics, and Computational Biology, Virginia Tech, Blacksburg, Virginia, USA; b Department of Civil and Environmental Engineering, Virginia Tech, Blacksburg, Virginia, USA; c Fralin Life Science Institute, Blacksburg, Virginia, USA; d Department of Computer Science, Virginia Tech, Blacksburg, Virginia, USA; The University of Tokyo

**Keywords:** antibiotic resistance, bacteriophages, insertion sequence, integrative elements, metagenomics, mobile genetic elements, mobilome, plasmids, transposons

## Abstract

Bacterial mobile genetic elements (MGEs) encode functional modules that perform both core and accessory functions for the element, the latter of which are often only transiently associated with the element. The presence of these accessory genes, which are often close homologs to primarily immobile genes, incur high rates of false positives and, therefore, limits the usability of these databases for MGE annotation. To overcome this limitation, we analyzed 10,776,849 protein sequences derived from eight MGE databases to compile a comprehensive set of 6,140 manually curated protein families that are linked to the “life cycle” (integration/excision, replication/recombination/repair, transfer, stability/transfer/defense, and phage-specific processes) of plasmids, phages, integrative, transposable, and conjugative elements. We overlay experimental information where available to create a tiered annotation scheme of high-quality annotations and annotations inferred exclusively through bioinformatic evidence. We additionally provide an MGE-class label for each entry (e.g., plasmid or integrative element), and assign to each entry a major and minor category. The resulting database, mobileOG-db (for mobile orthologous groups), comprises over 700,000 deduplicated sequences encompassing five major mobileOG categories and more than 50 minor categories, providing a structured language and interpretable basis for an array of MGE-centered analyses. mobileOG-db can be accessed at mobileogdb.flsi.cloud.vt.edu/, where users can select, refine, and analyze custom subsets of the dynamic mobilome.

**IMPORTANCE** The analysis of bacterial mobile genetic elements (MGEs) in genomic data is a critical step toward profiling the root causes of antibiotic resistance, phenotypic or metabolic diversity, and the evolution of bacterial genera. Existing methods for MGE annotation pose high barriers of biological and computational expertise to properly harness. To bridge this gap, we systematically analyzed 10,776,849 proteins derived from eight databases of MGEs to identify 6,140 MGE protein families that can serve as candidate hallmarks, i.e., proteins that can be used as “signatures” of MGEs to aid annotation. The resulting resource, mobileOG-db, provides a multilevel classification scheme that encompasses plasmid, phage, integrative, and transposable element protein families categorized into five major mobileOG categories and more than 50 minor categories. mobileOG-db thus provides a rich resource for simple and intuitive element annotation that can be integrated seamlessly into existing MGE detection pipelines and colocalization analyses.

## INTRODUCTION

Bacterial mobile genetic elements (MGEs) are of broad interest across multiple research communities. MGEs are critical drivers of horizontal gene transfer (HGT; i.e., the movement of genetic material between nonparental lineages of bacteria) (1, 2). MGEs are especially a concern as a fundamental driver of the spread of antibiotic resistance. Antibiotic resistance is a growing global threat to public health (3), empowering bacterial infections to survive antibiotic treatment and rendering these life-saving drugs ineffective. For instance, the multidrug resistance plasmid NR1 (R100), first recovered from a Shigella flexneri isolate in the 1950s (4), was found to confer resistance through its carriage of a mobile transposon (Tn*21*) harboring multiple antibiotic resistance genes (ARGs) (2, 5). Antibiotic resistance is now pandemic in many clinically relevant bacteria and pathogens enriched with ARG-harboring MGEs ranked among the most urgent and serious threats identified in the 2019 U.S. Centers for Disease Control and Prevention’s Antibiotic Threats report (6). Thus, surveilling the occurrence of HGT in genomic data is a key tool in the fight to abate the global spread of antibiotic resistance. Unfortunately, a major hinderance to research centered on MGEs is that currently available MGE databases are disparate, redundant, and contain extraneous genes that encode accessory functions for the host bacterium.

Next generation sequencing is commonly applied to profile MGEs in environmental or clinical samples (e.g., shotgun metagenomics or whole-genome sequencing) using bioinformatic analysis to identify molecular MGE signatures, such as genes encoding recombinases (7–11), sequence features (12), or even the full nucleotide sequence of MGEs (13, 14). However, unlike ARGs, which have benefitted from widespread efforts to curate unified databases (e.g., the Comprehensive Antibiotic Resistance Databases [CARD] [15] or the Structured Antibiotic Resistance Gene Database [SARG] [16]), collections of MGEs have thus far been compiled in disparate, independent databases. This situation has led to redundant entries, inconsistent nomenclature, and the presence of extraneous genes. Furthermore, there is no centralized resource for MGE hallmark genes that could serve as the basis for annotating diverse classes of MGEs. Instead, decentralized databases exist for phages, including pVOG (17) and the GutPhage Database (GPD) (18); insertion sequences, ISfinder (19); integrative genomic elements (IGEs), ICEberg (11), immedb (20); or plasmids, COMPASS (21), NCBI Plasmid RefSeq (22). While ACLAME (23) combines multiple element types, there has not been a substantial update to this database since 2010 (23, 24).

**FIG 1:**
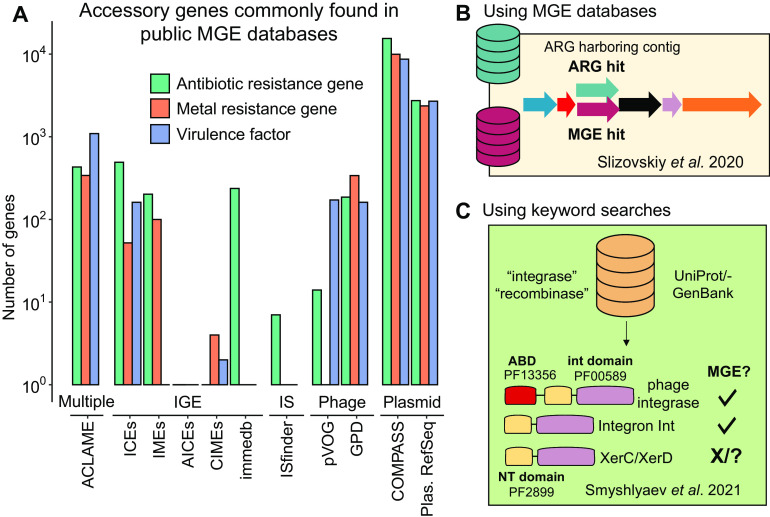
Challenges associated with existing databases for MGE colocalization analysis. (A) Example accessory genes found within MGE databases, with the databases grouped by the element class that they are intended to contain. Annotation criteria and databases are provided in Supplementary Methods. ACLAME is a database of multiple element types, thus is labeled “multiple.” (B) Synthesis of Slizovskiy et al. (10). Overlap in MGE/ARG databases produces ambiguous annotations of mobile ARGs. (C) Synthesis of Smyshlyaev et al. (38). The largest phylogenetic subgroup of tyrosine recombinases includes the *xerC/xerD* family of simple tyrosine recombinases, meaning studies using keyword search-based databases are likely to inadvertently include false-positive sequences. IGE, integrative genomic elements; IS, insertion sequence.

Current databases of MGEs also contain accessory genes, such as ARGs (5, 25), metal resistance genes (5, 26), or virulence factors (1, 27–29), which are irrelevant for element classification and may themselves be separate targets in genomic analyses (30–32) (Fig. 1A). For instance, Slizovskiy et al. found that overlap in MGE and ARG databases resulted in ambiguous or even erroneous annotations of mobile ARGs (10) (Fig. 1A and B). Well-documented accessory genes such as ARGs can be removed using existing databases (Fig. 1A); however, many cargo genes with tenuous relevance to mobility will remain. Thus, the presence of these cargo genes in MGE databases leads to the frequent occurrence of false-positive matches that confound and complicate MGE annotation (10) (Fig. 1B). To combat this, other studies have used custom databases of MGE-marker genes created using keyword searches against public databases such as UniProt or GenBank (e.g., integrase, recombinase, or transposase) (7–9, 25, 33, 34). While providing a solution to the presence of accessory genes, such usage of large-scale public repositories may introduce poorly characterized or moonlighting (i.e., multifunctional) members of MGE protein families (35–37). For instance, Smyshlyaev et al. demonstrated that the largest phylogenetic subgroup of tyrosine recombinases, a large protein family including phage and integron integrases, included the *xerC/xerD* family of simple tyrosine recombinases, which are infrequently associated with MGEs (38) (Fig. 1C). Further, there is increasing recognition of the need to classify MGEs, especially plasmids, using the entire backbone of the element rather than on traditional markers such as replicons and mobilases (11, 21, 39, 40). However, which genes might suitably comprise the “backbone,” or essential and conserved regions of MGEs, remains uncertain. Thus, there is a need for a structured resource and classification system that limits false positives, provides reliable matches to well-characterized MGEs, and centralizes available knowledge pertaining to diverse MGE classes.

To facilitate MGE annotation, we developed the mobile orthologous groups database (mobileOG-db), an interactive resource compiling a comprehensive variety of proteins that mediate the essential functions or “life cycle” of bacterial MGEs. Here, we define the essential functions of MGEs to include: (i) integration and excision (IE) from one genetic locus to another; (ii) replication, recombination, or nucleic acid repair (RRR); (iii) interorganism transfer (T); (iv) element stability, transfer, or defense (STD); and (v) phage (P) specific biological processes (e.g., genome packaging, or lysis and lysogeny).

The motivation of this work was to provide a structured and central resource for MGE annotation that is rooted in a biologically defensible classification of MGE protein function. In support of this effort, we analyzed 10,776,849 proteins from eight databases containing MGEs to: (i) remove accessory or poorly characterized genes; (ii) classify each entry as belonging to a phage, plasmid, insertion sequence, or IGE; and (iii) assign each entry a major mobileOG category (i.e., IE, RRR, T, STD, P) and one of over 100 minor mobileOG categories. We posit that these proteins are suitable candidate MGE hallmarks because of the key functions they perform, and thus they can be used for accurately detecting and characterizing MGEs in various genomic data sets.

## RESULTS

**FIG 2:**
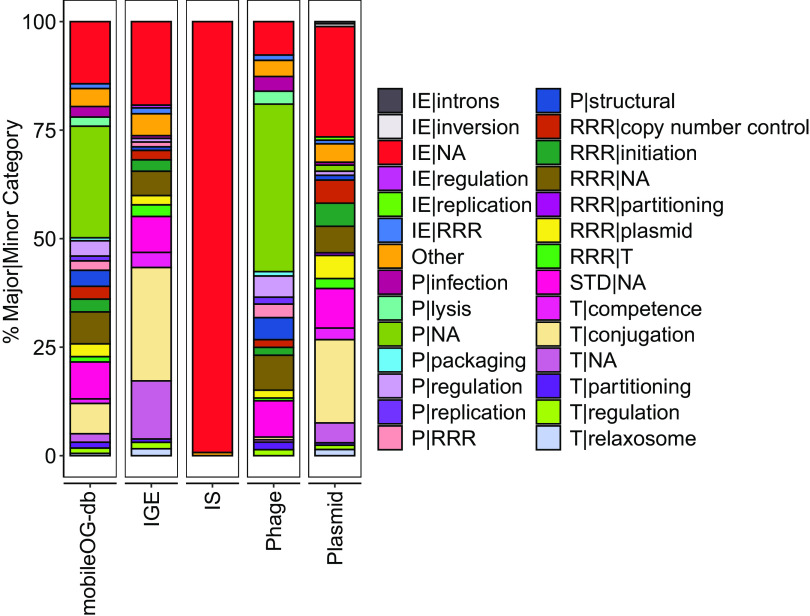
mobileOG-db comprises a diverse repertoire of proteins that orchestrate the unique life cycles of integrative genomic elements (IGEs), insertion sequences (IS), bacteriophages, and plasmids. Manually ascribed major and minor categories of entries derived from phage, plasmid, IS, or IGE sequences are displayed. The key displayed has the form Major mobileOG category|Minor mobileOG Category. Only categories with >1% occurrence in a given element type were included for visualization.
- More than 50 minor mobileOG Categories (Table S4), including: initiation (a subcategory of RRR referring to the Rep family proteins), structural or infection (subcategories of P), shufflon, inversion, or intron (subcategories of IE), conjugation, competence, or phage receptor (subcategories of T), among others (Fig. 2; Table S4).
- Element class labels derived from the source databases of phage, plasmid, insertion sequence, IGE, or multiple.

Here, we analyzed 10,776,849 proteins derived from eight databases containing bacterial MGEs to create a novel database, mobileOG-db, that directly addresses limitations imposed by current resources. Through an iterative annotation process, we eliminated 8,728,599 protein sequences from this set that were uncharacterized or indefensible as candidate MGE hallmarks from a biological standpoint. Because each sequence is directly derived from an existing MGE database, we reduce the probability of false positives incurred because of multifunctional or moonlighting proteins present in databases like UniProt. In addition, we provide a classification system consisting of:

- Major mobileOG categories: IE, STD, P, RRR, T (Fig. 2).

**FIG 3:**
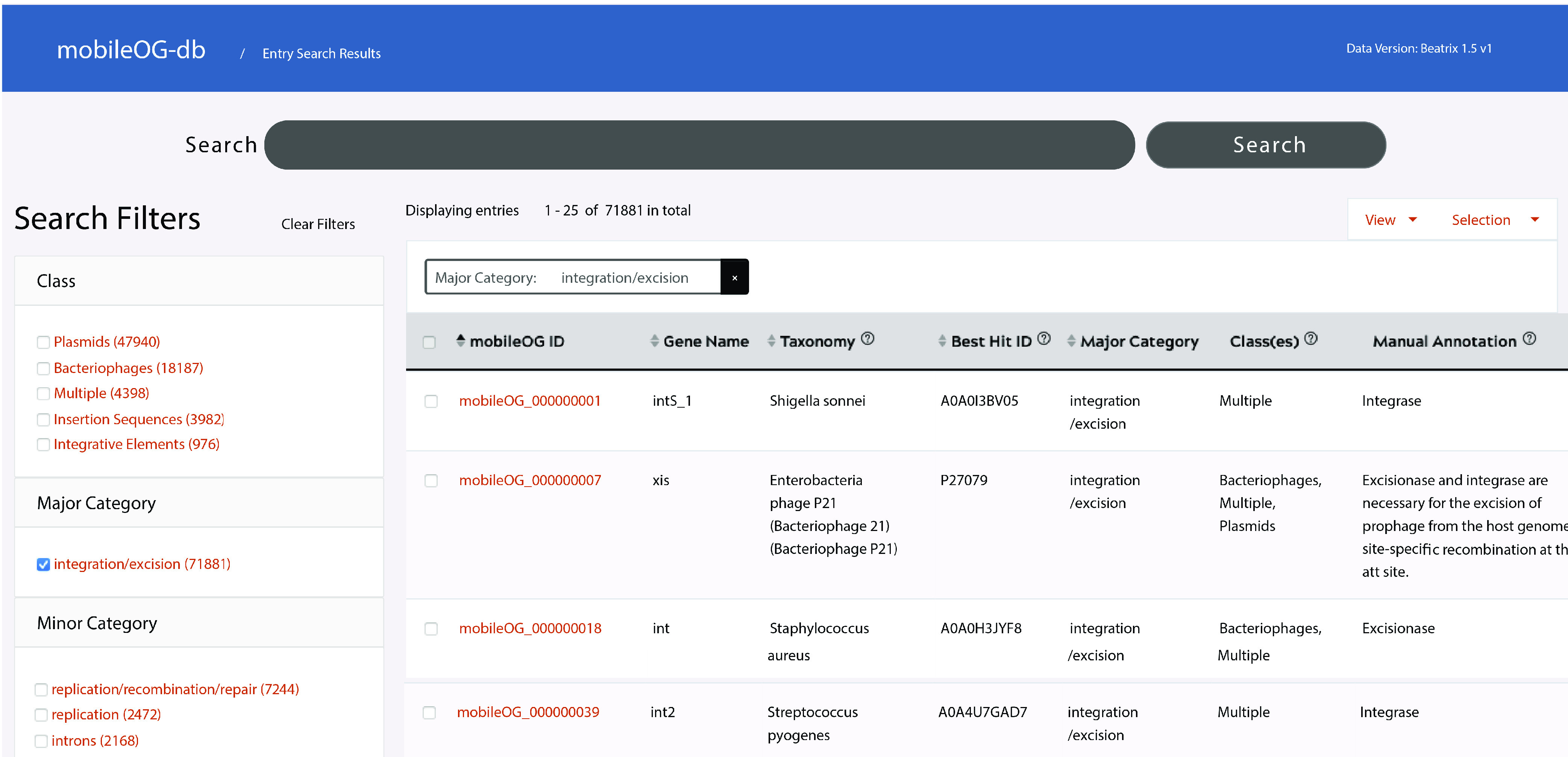
The mobileOG-db web-interface provides an interactive format for parsing mobileOG-db entries. mobileOG entries, representing deduplicated sequences, can be manually selected by category or by element type (e.g., plasmid, bacteriophage, or insertion sequence).

Altogether, this first release (beatrix) of mobileOG-db comprises 775,257 deduplicated proteins, including 29,721 derived directly from manually curated entries; 6,346 protein clusters or families (defined as greater than 40% identical over 50% of the subject and query length; see Materials and Methods); 2,444 unique manual annotations, and 1,393 references. The mobileOG-db web interface (Fig. 3) provides a simple and intuitive way to work with the database. Through this interface, users are offered the opportunity to identify overlap between element class gene contents, download custom databases, or select only manually curated or homologous entries. The complete database (comprising keyword search results, manually curated, and homology-based entries) is available as a standalone download on the website, while the web interface exclusively hosts the manually curated sequences and their homologs (Fig. 3).

In addition to aggregating and classifying existing databases, the curation of diverse MGE protein sequences allowed for the reannotation of more dated public database entries in light of recent discoveries. For example, we found components of several distinct MGE-defense systems on diverse element types (Table S5). These include the Bacteriophage Exclusion (BREX) (41) system genes *brxB*, *brxF*, *pglX*, *brxL*, among others (Table S5). Surprisingly, *pglX* gene homologs as well as the cognate antitoxin were found across several element types, including phages in the Gut Phage Database (Table S5), while components of the cyclic oligonucleotide-based antiphage signaling system (CBASS) (42) were exclusively found within ICEs and plasmids (Table S5). Further, we note the presence of several homologs of CRISPR-system components encoded on ICEs, plasmids, and even bacteriophages in the GutPhage database (Table S5). While the functionality of these proteins is unknown, the presence of defense system components highlights the potential for yet unexplored MGE cross talk that may contribute to interelement or interelement class (e.g., plasmid versus phage) competition. The accessions of these sequences are available as standalone downloads in Table S5 and are incorporated into mobileOG-db under the STD major category.

### Usage recommendations and examples.

For detecting and classifying elements from long genomic segments (e.g., long reads or assembled short reads), it is recommended that multiple colocalized hits in close proximity should be incorporated into the annotation criteria, similar to the pattern-based colocalization approach leveraged by ICEBerg (11) for IGE detection. Likewise, because plasmids and phages frequently encode homologs of RRR machinery that are also present in exclusively cellular DNA, it is noted hits solely to RRR modules are not necessarily indicative of an MGE (43). An additional caveat is hits to type 4 secretion systems may not be indicative of an MGE; paralogues of these proteins are also virulence determinants in some organisms (44). A simple annotation pipeline, mobileOGs.pl-kyanite (https://github.com/clb21565/mobileOG-db/tree/main/mobileOG-pl), has been developed (Supplementary Methods; Table S5) to allow for automated element annotation (Supplementary Methods; Fig. S2) that generates, annotates, and identifies orfs with homology to mobileOGs. Then, using a python script, it produces a summary table for the number of mobileOGs of specific element classes (e.g., phage or plasmid) were found for the contig. Using mobileOGs.pl-kyanite, we evaluated the ability of mobileOG-db annotations to correctly label sequences from COMPASS or pVOG as plasmids and phages, respectively (Table S2). This pipeline enabled successful classification of up to 98.2% and 99.7% of the plasmids and phages, respectively (https://doi.org/10.6084/m9.figshare.15170736; Fig. S2; Table S3). Importantly, we acknowledge that mobileOG-db alone cannot provide a highly granular classification of MGEs, as this would require leveraging additional sequence features, including, for example, recombination or replication initiation sites. However, database-derived annotations display strong agreement with VirSorter on real-world data (Fig. S2 and 3).

## DISCUSSION

While the goal of this work was to establish a well-curated MGE database, future research can further refine approaches to harness this information, depending on the application. In addition, though mobileOG-db addresses many of the limitations of existing resources for MGE annotation, particularly for colocalization applications, it is not a replacement for databases of full-length MGEs, e.g., PLSDB (45), TnCentral (46), and ISfinder (19). The creation of mobileOG-db relied on existing documentation of protein family function in the literature. Thus, proteins lacking experimental characterization in the function of MGE biology are likely absent from mobileOG-db. On the other hand, mobileOG-db also offers the distinct advantage over databases of individual elements in that it provides a multiclass labeling scheme derived from the databases of origin (Fig. 2). This structure allows for analysis of MGEs as hierarchical entities which would otherwise be impossible when using databases of specific MGE classes. To ensure long-term utility of the database, mobileOG-db will be updated approximately once a year with major changes, bug fixes, and new annotations. Minor grammatical or technical errors will be fixed on a semiregular basis as discovered or reported through the GitHub page (https://github.com/clb21565/mobileOG-db) or through the “Contact us” feature of the mobileOG-db website.

The rapidly increasing volume of sequence data has outpaced the ability to annotate and classify MGEs, in part due to a lack of a suitable knowledgebase (39). Thus, mobileOG-db was created specifically to address this critical gap. mobileOG-db and its web interface provide the ability to test a targeted hypothesis with reliable, customized databases tailored to the research objective. For instance, users interested in detecting mobile ARGs could select insertion sequence proteins, transfer/conjugation proteins, and plasmid-associated proteins (e.g., RepA, FinO), thus allowing for the detection of conjugative plasmid bearing insertion sequences, a key mechanism of ARG HGT (47). In the future, we expect the structured language provided by mobileOG-db will enable novel analyses that leverage the diverse and compositional nature of MGEs to enhance and refine the profiling of the dynamic mobilome. Such efforts will advance a diverse array of critical research fronts, particularly efforts aiming at understanding and tracking the spread of antibiotic resistance.

## MATERIALS AND METHODS

### Filtering accessory and uncharacterized MGE proteins from public databases.

Our curation efforts aimed to remove proteins with accessory or poorly understood functions from the contents of ICEBerg 2.0 (11), COMPASS (21), NCBI Plasmid RefSeq (22), GutPhage Database (18), pVOG (17), ISfinder (19), ACLAME (23), and immedb (20). To focus curation efforts exclusively on those referenced in the MGE literature, we created and queried a database of MGE abstracts from PubMed. To build this database, we gathered a set of descriptive keywords related to MGEs (Table S1) referencing *Mobile DNA III* (48) to identify important keywords. These terms were searched against NCBI PubMed using entrez to extract article metadata and abstracts. To exclude unrelated abstracts, the initial set was filtered to remove those with less than three keyword matches. The final resulting abstract set was then queried in subsequent searches and manually curations.

**FIG 4:**
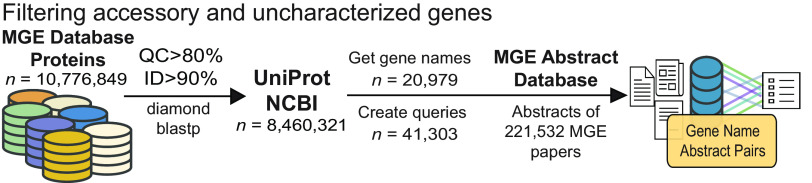
Filtering accessory genes using a database of published scientific abstracts relating to bacterial MGEs. The contents of the eight databases (*n* = 10,776,849) were mapped to UniProt entries and assigned gene names and annotations. The resulting gene names (*n* = 20,979) were converted into queries and searched against the MGE abstract database to create gene name abstract set pairs.

A pan-mobilome, i.e., an extensive collection of sequences comprising diverse MGEs, was created by merging the contents of eight publicly available MGE databases into a single database of protein sequences. The genomes comprising pVOG, COMPASS, and immedb, all nucleotide sequence databases, were processed with prodigal (v2.6.3) to generate open reading frames using the -p meta setting. This setting was chosen because the alternative, -p single, requires 20,000 bp of training data which would not be possible for many of the MGEs (49). These databases were selected as they comprise a representative sample of MGE diversity. Future iterations of mobileOG-db will expand to other databases to improve the coverage of MGE protein functional diversity. The final aggregated data set included 10,776,849 sequences (Fig. 4). The 10,776,849 proteins were searched against UniProt (release-2020_06) using diamond (50) blastp, with minimum identity 90% and minimum query coverage of 80%. Of the 10,776,749 sequences, 8,460,321 had matches to UniProt that passed filtering criteria, likely due to erroneous or truncated open reading frames generated by prodigal. Gene names for the 8,460,321 protein sequences were then extracted from a merged Bacterial, Archaeal, and viral UniProt knowledge base (.dat file downloaded using wget from the UniProt ftp server) with a custom script (available on the project GitHub page, https://github.com/clb21565/mobileOG-db/tree/main/scripts). This process produced 110,234 gene names corresponding to the 8,460,321 sequences; 20,979 of the 110,234 gene names were unique. Many of the resulting gene names were likely to create spurious hits (e.g., antiholin gene *S*, UniProt entry P03705), or contained additional characters (e.g., *traG_2*, UniProt entry G9G740), and so the names were processed to produce suitable queries for searching against the abstract database. Queries were produced from the 20,979 gene names in the following way: Gene names with three or fewer characters were prefixed with “protein” or “gene” to reduce spurious hits. Gene names with an underscore or special character were split on either side (e.g., *tnpA_2* would become two queries: *tnpA* and *2*) and both sides were searched against the MGE-abstract database. Altogether this process produced 41,303 unique queries (the complete list of search terms is available on the figshare project site: https://doi.org/10.6084/m9.figshare.15170736). These queries were then searched against the MGE abstract database, resulting in 7,012 gene name-abstract set pairs that were then passed to manual curation.

### Manual curation and classification of MGE protein family function.

**FIG 5:**
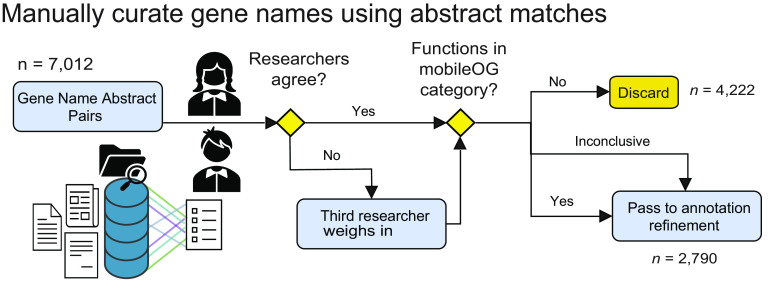
Preliminary gene name curation. A total of 7,012 gene abstract-set pairs were manually inspected by at least two researchers to determine whether the gene name could belong to a protein that performed one of the target processes. This curation resulted in 4,222 discarded entries (identified as likely cargo, or off-target matches) and 2,790 gene name-abstract set pairs to have refined functional annotations.

**FIG 6:**
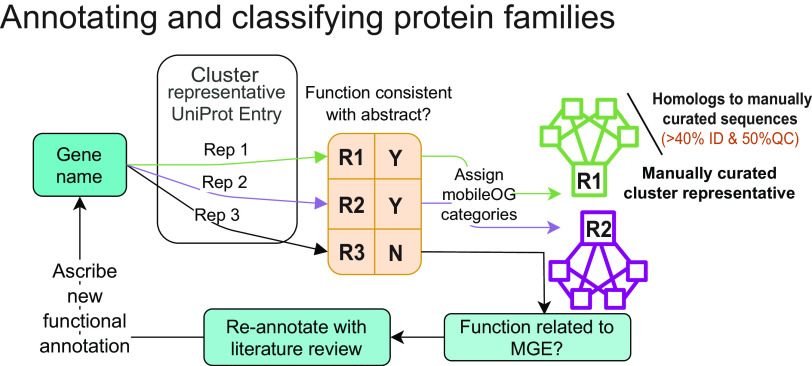
Annotation refinement and classification of MGE protein sequences. Each of the 2,790 gene names passed to refinement typically belonged to multiple protein clusters or families. For each cluster, one named representative was selected, and its putative function was compared with the literature-derived descriptions recovered from the abstract analysis. If the UniProt/NCBI entry did not support a link between the gene name and function, the protein was annotated via literature review by one of two researchers, with uncertainties and disagreements settled by discussion. Protein families that were ultimately confirmed to perform a target function were assigned a major and minor mobileOG category.

Each of the 7,012 gene name-abstract set pairs were scanned by at least two researchers to determine whether the gene name might encode a protein that performed one of the target functions (IE, T, P, RRR, or STD) (Fig. 5). This process resulted in the discarding of 4,222 gene names that corresponded to proteins with accessory or unknown function, leaving 2,790 gene names for annotation refinement. These 2,790 gene names corresponded to 98,465 sequences, with many sequences sharing the same name (see Supplementary Methods for examples). To reduce the extent of curation required, these proteins were grouped into protein clusters, or families defined as being 40% identical over 50% of the reference and query sequence using mmseqs2 (mmseqs easy-clust -c 0) (51). This produced 13,441 clusters associated with the 2,790 names. This coverage criterion was selected to reduce the incidence of singlet clusters induced by fragmented open reading frames. The 40% identity cut-off is used by the enzyme commission to determine whether a protein fold retains the same function as a reference fold (52). One named representative from each cluster (i.e., a cluster representative bearing one of the 2,790 gene names) was selected and passed to annotation refinement (Fig. 6). To refine annotations and assign mobileOG categories, the cluster representative’s putative function was compared with that described by the abstracts. If it was inconsistent with the attributing abstract(s) (see Supplementary Methods), the sequence was reannotated following a review of the literature recovered by searching for the gene name and putative function in PubMed. Finally, curated cluster representatives were assigned a major and minor mobileOG category. Minor mobileOG categories were assigned as secondary functional labels under the major mobileOG category by considering what putative function the family was associated with. Homologs of the manually curated sequences were assigned a category using the manually curated representative(s) (Fig. 6).

To improve coverage, keyword matches in the fasta headers with a table of MGE protein keywords (Table S2) were used as evidence for inclusion in mobileOG-db. The evidence used to determine inclusion (manual curation, homology, or keyword searches) is recorded in mobileOG-db. Examples of the rationales applied are provided in Supplementary Methods. Sequences with matches to Swiss-Prot entries, which are manually curated UniProt entries, were considered a special case and were manually curated regardless of whether they were returned during the abstract analysis. Final mobileOG-db entries are deduplicated at 100% identity using mmseqs2 (–min-seq-id 1 -c 1 –cov-mode 0), and the occurrence of identical sequences across different databases and element classes is recorded (Fig. 6). Orthology assignments from EggNOG (53) as well as associated pfam (54) domain labels are provided through the web interface and as standalone downloads. The gene names, queries, and the abstract database, are available at the Figshare project (https://doi.org/10.6084/m9.figshare.15170736).

### Data availability.

mobileOG-db is available at mobileogdb.flsi.cloud.vt.edu/, where users can browse, filter, search, and download customized data sets and references. Scripts used in the text mining analysis are available at https://github.com/clb21565/mobileOG-db/tree/main/scripts, while the preliminary annotation pipeline, mobileOG-pl. kyanite can be accessed at https://github.com/clb21565/mobileOG-db/tree/main/mobileOG-pl.
