# Supplementary material for: mobileOG-db: a Manually Curated Database of Protein Families Mediating the Life Cycle of Bacterial Mobile Genetic Elements
Source: Appl Environ Microbiol. 2022 Aug 29;88(18):e00991-22. doi: 10.1128/aem.00991-22 (PMC9499024; doi:10.1128/aem.00991-22)
Supplement: Supplemental file 1 — Supplemental methods, Fig. S1 to S3, and Tables S1 and S2. Download aem.00991-22-s0001.pdf, PDF file, 0.4 MB [file aem.00991-22-s0001.pdf]

mobileOG-db: a manually curated database of protein families mediating the life cycle of bacterial mobile genetic elements

SUPPLEMENTAL METHODS

(i) Annotation of accessory genes in mobile genetic element databases.
(ii) Example rationale for annotating proteins

Figure S1. Example of incorrect annotation manually reconciled in mobileOG-db.

(iii) Description of the mobileOG-kyanite for autonomous element detection and classification

Figure S2. Description of mobileOG.pl-kyanite, a preliminary pipeline for autonomous element detection and classification.

SUPPLEMENTARY DATA

Table S1. Keywords used to identify mobile genetic element abstracts in PubMed.

Table S2. Keywords and their associated categories created to identify putative MGE sequences that are associated with the target categories in the merged database.

Table S3. Evaluation of mobileOG-kyanite, a pipeline for identifying putative mobile element contigs. Attached as csv.

Table S4. Complete list of major and minor mobileOG category combinations. Attached as csv.

Table S5. CRISPR, BREX, and CBASS anti-phage system components present within mobileOG-db. Attached as csv.

Figure S3. Comparison of mobileOG-db.pl in classifying putative phages and prophages derived from wastewater metagenomes described in Brown & Keenum et al 2021 [1]. Top panel: VirSorter produces three levels of confidence for the annotation of phages in metagenomic data with different levels of confidence in the prediction. "Confident phage" refers to the highest level of confidence in the VirSorter (category-1); confident prophage corresponds to category 4 (the highest-confidence of a positive prophage identification); and "Likely phage" refers to category-2 (a "medium" level of confidence in phage identification). "Conservative Plasmids" refers to a more stringent cut-off selected in the mobileOG-db pipeline (k= 15 and purity $\geq$ 80%). Bottom panel: protein-coding gene content is consistent with a tentative annotation as plasmid fragments.

Supplemental Methods

(I) Annotation of accessory genes in public mobile genetic element databases.

Antibiotic resistance genes, metal resistance genes, and virulence factors were identified in public databases using diamond blastp [2], with cut-offs of >90% sequence identity and >80% query coverage. Antibiotic resistance genes were annotated using CARD v. 3.0.7 [3]; metal resistance genes were annotated using BacMet [4], and virulence factors were annotated using VF-db [5].

(II) Example rationale of protein annotations.

Protein families were included in mobileOG-db only if there was experimental evidence of their direct involvement with one of the targeted functions. Protein families with only indirect interactions with one of the target functions were not included unless they had been shown to be essential for element

persistence or replication. For example, these criteria excluded ribonucleotide reductases found within many phage genomes [6], which only have an indirect impact on replication through nucleotide metabolism [7,8], except under conditions of anaerobic growth [7,9]. While these proteins are useful indicators of phage diversity [10,11], we were unable to find evidence of a direct role in replication other than nucleotide metabolism and thus these proteins are not present in mobileOG-db. By contrast, phage-encoded thymidylate synthase homologs provide nucleotide substrates for replication and control levels of methyl- or hydroxymethyl- thymidine monophosphates [12]. These modified pyrimidines can then be further hypermodified [13] by additional functional moieties [12,14], which alter the steric properties of the nucleic acid of the viral genome. This process can therefore provide a phage genome with defense against host-encoded CRISPR [15] and restriction modification systems [16–19]. Thus, thymidylate synthases were included in mobileOG-db and categorized in the replication/recombination/repair major category with minor categories stability and defense.

By contrast, we found that there were several examples of proteins with names that did not match the results of the abstract database, and therefore had to be manually curated to reconcile the disagreement. For example,

> tr|A0A2Z2Q3C7|A0A2Z2Q3C7_9RHIZ Polyamine ABC transporter ATP-binding protein OS=Agrobacterium larrymoorei OX=160699 GN=repB PE=3 SV=1

The protein repB was identified as a regulator of plasmid replication by the abstract analysis and this sequence initially appeared to be an erroneous attribution of the name, or a protein with the same name but different function. Upon further inspection, it became apparent that the header was not descriptive of the putative function of the protein:

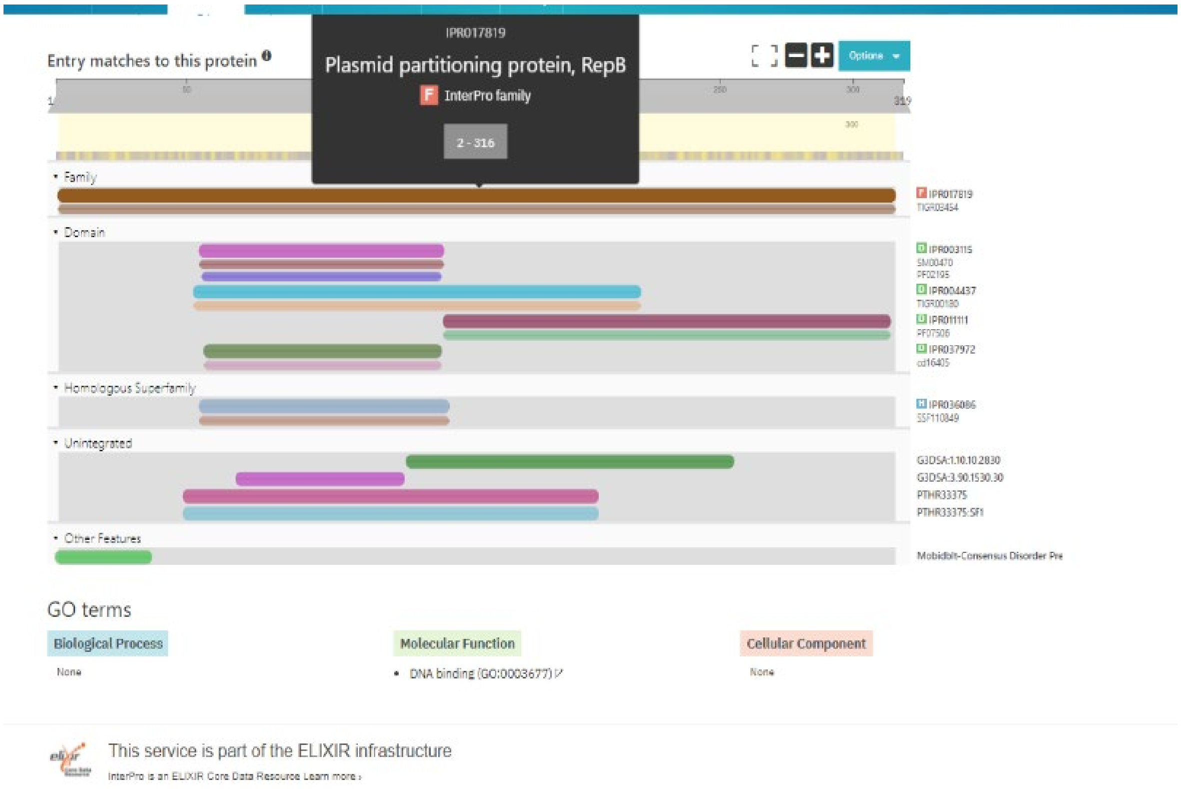

View protein in InterPro
IPR004437, ParB/RepB/Spo0J
IPR003115, ParB/Sulfiredoxin_dom
IPR036086, ParB/Sulfiredoxin_sf
IPR017819, Plasmid_partition_RepB
IPR011111, Plasmid_RepB
IPR037972, RepB_N

Figure S1. Example of incorrect annotation manually reconciled in mobileOG-db.

Thus, this entry was included in the manually curated sequences as it had a positive association between name, literature, and putative function. UniProt was additionally contacted to seek a correction for this entry.

Below are two examples of MGE gene names that also correspond to names of other genes and proteins. *mobC* is also the name of a gene encoding a mobilase associated with conjugal plasmid transfer [20]; *motA* also refers to a gene encoding a T4 phage transcriptional regulator [21].

tr|A0A0K2CS33|A0A0K2CS33_CITFR Molybdopterin-guanine dinucleotide biosynthesis protein mobc OS=Citrobacter freundii OX=546 GN=mobC PE=4 SV=1

tr|A0A174YTE7|A0A174YTE7_9FIRM Chemotaxis protein MotA OS=[Eubacterium] eligens OX=39485 GN=motA PE=4 SV=1

(ii) mobileOG-db.pl-kyanite, a preliminary pipeline to detect and classify genomic contigs or long reads as putative MGEs.

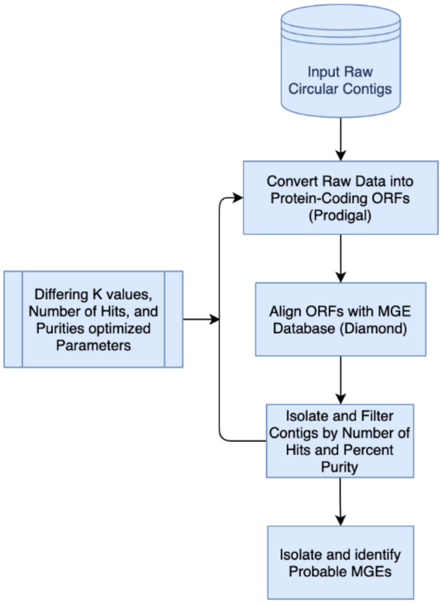

Figure S2. mobileOG-db.pl-kyanite takes genomic contigs as input, converts the nucleotide sequences to open reading frames using prodigal, then aligns the open reading frames against mobileOG-db. Different diamond settings can be used, and were tested for recovering phages or plasmids from a test data set.

SUPPLEMENTARY DATA

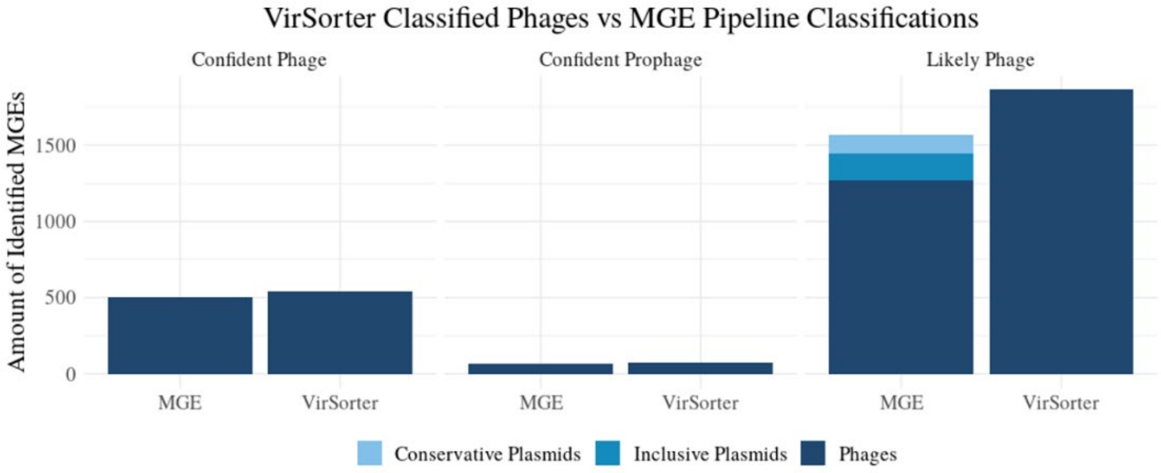

Figure S3. Comparison of mobileOG-db.pl-kyanite in classifying putative phages and prophages derived from wastewater metagenomes described in Brown & Keenum *et al*. 2021 [1]. **Top panel:** VirSorter [22] produces three levels of confidence for the annotation of phages in metagenomic data with different levels of confidence in the prediction. “Confident phage” refers to the highest level of confidence in the VirSorter (category-1); confident prophage corresponds to category 4 (the highest-confidence of a positive prophage identification); and “Likely phage” refers to category-2 (a “medium” level of confidence in phage identification). “Conservative Plasmids” refers to a more stringent cut-off selected in the mobileOG-db pipeline (k= 15 and purity $\geq$ 80%).

Table S1. Keywords used to identify MGE abstracts.

Keyword
competence
CRISPR
nuclease
replication
toxin
antitoxin
addiction
transposition
replication
DNA
capsid
tape measure
terminase
tail collar
baseplate
Reverse transcriptase
resolvase
invertase
shufflon
restriction
methyltransferase
mobile genetic element
transposon
integrative conjugative element
chromosomal integrative mobile element
mobile DNA
virus
prophage
phage
plasmid
incompatibility group
mobile
selfish genetic element
casposon
viral
proviral
insertion sequence
restriction modification
pINC
ICEBerg
mobilome
excision

integration
recombination
transposable element

Table S2. Keywords used to recover MGE functional proteins from the merged database.

Category	Include†	Do not include††
phage,structural	head,neck,capsid,baseplate,vertex,whisker,tail, sheathe,portal,coat,spike,neck,tape measure,virion,base plate,Tape-measure,Plate protein	conjugation,type VI secretion system,cytochrome c oxidase,two-tailed,cluster,conjugal,photosystem II stability,hammerhead,pilus,conjugative
phage,lysogeny	lysin,autolysin,endolysin,lysozyme,holin,antiholin,spanin,abortive infection	lysozyme if no "phage" or "virus"; hemolysin, haemolysin,choline,Lysinibacillus, hydrolysing
phage,regulation	regulatory cii,prophage repressor,tapemeasure,antirepressor,anti-repressor,phage late control	
phage,replication,packaging	terminase,terl	interleukin
integration,excision	integration,excision,integrase,tyrosine recombinase,serine recombinase,serine integrase,phage integrase,transposase,helper of transposition,excisionase,xis protein,cassette chromosome recombinase,Integration host factor,recombination directionality factor,shufflon,group I intron endonuclease,Tnp domain,Retron-type reverse transcriptase,intron endonuclease	chemotaxis
integration,excision,inversion	invertase,inversion	
integration,excision,replication,recombination,repair	resolvase	
stability,transfer,defense	addiction,toxin/antitoxin,antitoxin,YoeB,YoeF,HigB,CRISPR, toxin-antitoxin,RelE/ParE,entry exclusion,stbB,plasmid stabilization system,DNA methylase,restriction endonuclease,surface exclusion,restriction-modification,Protein kilB,kilB,Hok/Gef ,N-6-adenine-methyltransferase,N-6 DNA methylase,restriction enzyme,DNA adenine methylase	shiga toxin,Clavibacter michiganensis,michiganensis,RIGHA
transfer,conjugation	conjugation,pilus,conjugational,conjugative,type IV secretion system protein,mobilization,relaxase,mobilase,FtsK/SpoIIIE,FtsK,SpoIIIE,TraB,TraM,conjugal,VirB3,MobA/MobL,TrbC/VirB2	tram
CRISPR	CRISPR	
transfer,competence	competence	
replication,regulation	protein RepA, repZ, repL,	
phage,infection	adsorption,antireceptor,Super-infection exclusion	
replication,transfer,partitioning	ParB,RepB,Spo0J	
transfer	DNA transfer	

†Terms used as search queries.
††Search terms used to filter (remove) erroneous hits to a given category following the search.

Supplementary References

1. Brown CL, Keenum IM, Dai D, Zhang L, Vikesland PJ, Pruden A. Critical evaluation of short, long, and hybrid assembly for contextual analysis of antibiotic resistance genes in complex environmental metagenomes. Sci Rep [Internet]. Nature Publishing Group; 2021 [cited 2021 Feb 16];11:3753. Available from: http://www.nature.com/articles/s41598-021-83081-8

2. Buchfink B, Xie C, Huson DH. Fast and sensitive protein alignment using DIAMOND. Nat. Methods. Nature Publishing Group; 2014. p. 59–60.

3. Alcock BP, Raphenya AR, Lau TTY, Tsang KK, Bouchard M, Edalatmand A, et al. CARD 2020: antibiotic resistome surveillance with the comprehensive antibiotic resistance database. Nucleic Acids Res [Internet]. Oxford Academic; 2020 [cited 2021 Aug 14];48:D517–25. Available from: https://academic.oup.com/nar/article/48/D1/D517/5608993

4. Pal C, Bengtsson-Palme J, Rensing C, Kristiansson E, Larsson DGJ. BacMet: antibacterial biocide and metal resistance genes database. Nucleic Acids Res [Internet]. Oxford University Press; 2014 [cited 2021 Nov 1];42:D737. Available from: /pmc/articles/PMC3965030/

5. L C, D Z, B L, J Y, Q J. VFDB 2016: hierarchical and refined dataset for big data analysis--10 years on. Nucleic Acids Res [Internet]. Nucleic Acids Res; 2016 [cited 2021 Nov 1];44:D694–7. Available from: https://pubmed.ncbi.nlm.nih.gov/26578559/

6. Iyer LM, Anantharaman V, Krishnan A, Maxwell Burroughs A, Aravind L. Jumbo phages: A comparative genomic overview of core functions and adaptions for biological conflicts. Viruses [Internet]. Multidisciplinary Digital Publishing Institute (MDPI); 2021 [cited 2021 Jul 8];13. Available from: /pmc/articles/PMC7824862/

7. Lundin D, Torrents E, Poole AM, Sjöberg BM. RNRdb, a curated database of the universal enzyme family ribonucleotide reductase, reveals a high level of misannotation in sequences deposited to Genbank. BMC Genomics [Internet]. BioMed Central; 2009 [cited 2021 Jul 12];10:1–8. Available from: https://bmcgenomics.biomedcentral.com/articles/10.1186/1471-2164-10-589

8. Miller ES, Kutter E, Mosig G, Arisaka F, Kunisawa T, Rüger W. Bacteriophage T4 Genome. Microbiol Mol Biol Rev [Internet]. Microbiol Mol Biol Rev; 2003 [cited 2021 Jul 12];67:86–156. Available from: https://pubmed.ncbi.nlm.nih.gov/12626685/

9. Fontecave M, Mulliez E, Logan DT. Deoxyribonucleotide synthesis in anaerobic microorganisms: The class III ribonucleotide reductase. Prog Nucleic Acid Res Mol Biol. Academic Press; 2002;72:95–127.

10. Dwivedi B, Xue B, Lundin D, Edwards RA, Breitbart M. A bioinformatic analysis of ribonucleotide reductase genes in phage genomes and metagenomes. BMC Evol Biol [Internet]. BMC Evol Biol; 2013 [cited 2021 Jul 12];13. Available from: https://pubmed.ncbi.nlm.nih.gov/23391036/

11. Sakowski EG, Munsell E V., Hyatt M, Kress W, Williamson SJ, Nasko DJ, et al. Ribonucleotide reductases reveal novel viral diversity and predict biological and ecological features of unknown marine viruses. Proc Natl Acad Sci U S A. National Academy of Sciences; 2014;111:15786–91.

12. Weigele P, Raleigh EA. Biosynthesis and Function of Modified Bases in Bacteria and Their Viruses. Chem Rev [Internet]. 2016 [cited 2021 Jul 12];116:12655–87. Available from: https://pubs.acs.org/sharingguidelines

13. H.Gommers-Ampt J, Borst P. Hypermodified bases in DNA. FASEB J [Internet]. FASEB J; 1995 [cited 2021 Jul 12];9:1034–42. Available from: https://pubmed.ncbi.nlm.nih.gov/7649402/

14. Lee YJ, Dai N, Walsh SE, Müller S, Fraser ME, Kauffman KM, et al. Identification and biosynthesis of thymidine hypermodifications in the genomic DNA of widespread bacterial viruses. Proc Natl Acad Sci U S A [Internet]. Proc Natl Acad Sci U S A; 2018 [cited 2021 Jul 12];115:E3116–25. Available from: https://pubmed.ncbi.nlm.nih.gov/29555775/

15. Bryson AL, Hwang Y, Sherrill-Mix S, Wu GD, Lewis JD, Black L, et al. Covalent modification of bacteriophage T4 DNA inhibits CRISPRCas9. MBio [Internet]. American Society for Microbiology; 2015 [cited 2021 Jul 12];6. Available from: https://journals.asm.org/journal/mbio

16. Flodman K, Tsai R, Xu MY, Corrêa IR, Copelas A, Lee YJ, et al. Type II restriction of bacteriophage DNA with 5hmdU-derived base modifications. Front Microbiol [Internet]. Frontiers Media SA; 2019 [cited 2021 Jul 12];10:584. Available from: /pmc/articles/PMC6449724/

17. Miller PB, Wakarchuk WW, Warren RAJ. α-putrescinylthymine and the sensitivity of bacteriophage φW-14 DNA to restriction endonucleases. Nucleic Acids Res [Internet]. Nucleic Acids Res; 1985 [cited 2021 Jul 12];13:2559–68. Available from: https://pubmed.ncbi.nlm.nih.gov/2987859/

18. Huang LH, Farnet CM, Ehrlich KC, Ehrlich M. Digestion of highly modified bacteriophage DNA by restriction endonucleases. Nucleic Acids Res [Internet]. Oxford University Press; 1982 [cited 2021 Jul 12];10:1579–91. Available from: /pmc/articles/PMC320551/?report=abstract

19. Krüger DH, Bickle TA. Bacteriophage survival: multiple mechanisms for avoiding the deoxyribonucleic acid restriction systems of their hosts. Microbiol Rev [Internet]. Microbiol Rev; 1983 [cited 2021 Jul 12];47:345–60. Available from: https://pubmed.ncbi.nlm.nih.gov/6314109/

20. Garcillán-Barcia MP, Francia MV, De La Cruz F. The diversity of conjugative relaxases and its application in plasmid classification. FEMS Microbiol Rev. 2009. p. 657–87.

21. Schmidt RP, Kreuzer KN. Purified MotA protein binds the -30 region of a bacteriophage T4 middle-mode promoter and activates transcription in vitro. J Biol Chem. Elsevier; 1992;267:11399–407.

22. Roux S, Enault F, Hurwitz BL, Sullivan MB. VirSorter: Mining viral signal from microbial genomic data. PeerJ [Internet]. PeerJ Inc.; 2015 [cited 2021 Jun 30];2015:e985. Available from: https://peerj.com/articles/985
